# Smartphone Applications for Period Tracking: Rating and Behavioral Change among Women Users

**DOI:** 10.1155/2020/2192387

**Published:** 2020-09-01

**Authors:** Reema A. Karasneh, Sayer I. Al-Azzam, Karem H. Alzoubi, Suhaib M. Muflih, Sahar S. Hawamdeh

**Affiliations:** ^1^Department of Basic Medical Sciences, Faculty of Medicine, Yarmouk University, Irbid, Jordan; ^2^Department of Clinical Pharmacy, Faculty of Pharmacy, Jordan University of Science and Technology, Irbid, Jordan

## Abstract

**Background:**

The use of mobile apps for health and well-being has grown exponentially in the last decade, as such apps were reported to be ideal platforms for behavioral change and symptoms monitoring and management.

**Objective:**

This study aimed to systematically review period tracking applications available at Google Play and Apple App Stores and determine the presence, features, and quality of these smartphone apps. In addition, behavioral changes associated with the top 5 rated apps were assessed.

**Methods:**

This study used the Systematic Search Criteria through Google Play Store and iTunes Apple Store, using terms related to period tracking. Apps were scanned for matching the inclusion criteria and the included apps were assessed by two reviewers using the Mobile Application Rating Scale (MARS), a tool that was developed for classifying and assessing the quality of mHealth apps.

**Results:**

Forty-nine apps met the inclusion criteria. Most of the apps enabled setting user goals, motivations, and interactivity, tracking multiple symptoms or mood changes, allowed notifications, and used graphs to illustrate the tracking result over a specific period of time. The majority of features and functions within these apps were offered for free, while some apps included limited in-app purchases or needed Internet connection to function. Certain apps were reported by participants to promote behavioral change and increase knowledge and awareness regarding monthly periods.

**Conclusions:**

Period tracking apps were easy to use and navigate and can hence be readily adopted into routine tracking and management of periods. However, most apps were not based on significant evidence and may need further development to support period-related symptom management.

## 1. Introduction

The menstrual cycle is considered a biological marker that predicts women's general health [1]. Awareness of a woman about her own fertility is important for her to understand patterns of ovulation and menstrual cycle necessary for pregnancy planning or practicing contraception [2]. Monitoring ovulation time also provides a better understanding of women's own bodies and mental states and facilitates noticing early bodily symptoms such as the appearance of cervical secretions several days prior to ovulation [3].

Globally, mobile health apps were found to have a great impact on health behaviors by symptom monitoring and management utilizing a wide range of positive health outcomes. Moreover, mobile app use was reported to enhance the outcomes of several chronic illnesses and health issues [4–8]. For example, a meta-analysis of 1657 diabetic patients has shown 5% reduction in HbA1c values among those who used diabetes-related apps for self-management [7]. Positive results were also reported for autism, heart failure, weight management, smoking reduction, and overall lifestyle improvement. Additionally, such apps provided cost-effective, timely, and easily accessible methods for health promotion among different populations [9–11].

Mobile applications can be valuable tools to help women track their periods, as they offer effective features for self-care and symptom management. These features include reminders, graphic analysis, feedback, medications, health education, interactive questions, and connectivity to social media or their own social network [12–14]. In addition, they offer guidance on the use of contraceptives and information about their side effects, available services, and reminders of routine activities that enhance medication adherence [15]. Therefore, fertility awareness-based methods accessed by a mobile app without the requirement to interact with health professionals are considered a unique method that helps in tracking ovulation cycles and fertility symptoms [16]. Despite this, differences between these apps by effectiveness, overall quality, included features, and numerically using these features to reduce ovulation prediction errors have been scarcely reported [17]. In addition, consumers' decisions to select apps are steered by marketing jargon and users' reviews as there are no official or reliable quality markers [18]. Therefore, the aim of the current study was to perform a systematic review using a comprehensive search strategy to identify relevant smartphone apps related to period tracking, which are available at the App Store and Android Google Play Stores, and determine their composition and quality/effectiveness.

## 2. Methods

### 2.1. Systematic Search Criteria

Mobile apps were identified by searching the Apple iTunes and Android Google Play Stores. The following search terms were used: “period tracker,” “ovulation,” “menstrual,” and “fertility.” Each term was searched for in both App Stores listed during July 2019. The protocol of this study was approved by the institutional review board of the Jordan University of Science and Technology.

### 2.2. Eligibility Criteria and Selection of Apps

Following Preferred Reporting Items for Systematic Reviews and Meta-Analyses (PRISMA) guidelines, apps retrieved using the abovementioned search terms were downloaded onto respective devices of either iPhone or Android. Apps were included if they were available in both stores in English language, related to period tracking, and free to download. Primary screening was conducted independently by two reviewers to overcome the subjectivity in the assessment approach relying on the app's title. In cases were eligibility could not be determined, app description and photo previews presented by both stores were used for assessment.

### 2.3. Data Extraction

A detailed review of the included apps was conducted and data was independently extracted by the two reviewers including information on classification and objective and subjective qualities as described in the Mobile Application Rating Scale (MARS) [17, 19]. Classification of data included only characteristics of apps such as descriptive information on rating and technical aspects such as password protection, confidentiality, and security. Objective quality included engagement, functionality, aesthetics, and information features. The presence or absence of objective quality features was assessed in extracted information and if not found, then the feature was considered absent.

### 2.4. Quality Assessment

Apps quality criteria including engagement, functionality, aesthetics, and information quality in addition to subjective quality (satisfaction) items were assessed and scored for each included app using a 5-point Likert scale used in MARS. In the scale utilized, information quality was related to the accuracy of app description, goals, visual information, credibility, and evidence base of information provided. Furthermore, the overall mean of objective quality criteria for each included app in addition to the median score for each quality criteria was calculated [20].

### 2.5. Behavioral Change Assessment

Behavioral changes were associated with the top 5 ranked apps using an app-specific Behavioral Change MARS subscale [4]. The tool was developed on a web-based platform to facilitate the completion and collection of data. A link to the survey questionnaire was sent via an e-mail invitation to a convenience sample of potential participants, an e-mail invitation using available e-mail lists of employees at two major universities in Jordan. Females who accepted to participate in the study were asked to download and use the included period-related apps and to fill out the questionnaire that consisted of five questions related to behavior change (section G of MARS). Each of these questions assessed the impact of each app on women's awareness, knowledge, attitudes, intention to change, and help-seeking from a healthcare professional via online connection or by asking for a face-to-face appointment.

## 3. Results

**Figure 1:**
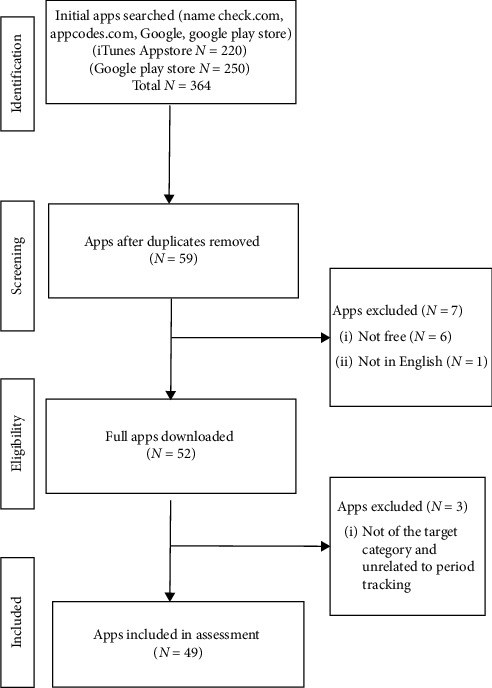
PRISMA diagram for iTunes and Google Play search results.

Figure 1 provides an overview of the selection process and categories for exclusion. Two hundred and twenty (*n* = 220) apps were initially identified from the Google Play Store and two hundred and fifty (*n* = 250) from Apple iTunes. Sixty apps (*n* = 60) remained after duplicates were removed: six (*n* = 6) of them were paid, one app (*n* = 1) was not in English language, and three apps (*n* = 3) were excluded because they were not of the target category and unrelated to period tracking. Therefore, forty-nine apps met the final inclusion criteria and were, thus, included in the study.

Most of the assessed apps enabled setting user goals, motivations, and interactivity, to be able to track multiple symptoms or mood changes, and simultaneously to allow the user to detect potential correlations between symptoms and onset of their period. The user can record symptoms, moods, vital signs, activities, nutrition, body temperature, blood pressure, and medications. Data were illustrated using a color-coding scheme and daily calendar format. Relevant data were also displayed in a line graph or chart to show daily and monthly inconstancies. Many apps were focused on helping women to schedule their ovulation and track fertility window (fertile days) and to follow the chance of getting pregnant with the ovulation predictor and predicted time of the next period. Other apps supported self-care maintenance in terms of recording daily health behaviors or including reminders for taking medications, drinking water, and dates of ovulation.

Characteristics of the included apps are shown in (Table 1). App Stores including Google Play Store and Apple Store offer a “star” rating system for available apps as a measure of app quality. Star rating for apps ranged from 1.8 to 5 (mean = 4.32). “Nabta Cycle” had the highest rating score “5” while *“DaysyView”* had the lowest score “1.8,” with one application “MyPeriod Tracker” without rating. Most of the apps (79%, *N* = 39) were updated in 2019. However, three of the assessed apps have not been updated in the last 4 years. “My Calendar-Period Tracker*”* and “Period Tracker-Period Calendar Ovulation Tracker*”* were the most downloaded apps “100.000.000+” while “Nabta Cycle” was the least “100+.”

### 3.1. MARS App Quality Scores

Table 2 shows the average of MARS scores between the two reviewers, the overall mean of objective quality score, and subjective quality scores for each of the included apps. Nabta Cycle app had the highest mean MARS score (4.475) followed by Femometer-Fertility Tracker (4.431), Period Tracker Flo, Ovulation Calendar & Pregnancy (4.418), Nyra-Period, Fertility & Ovulation Tracker App (4.370), and Maya-Period, Fertility, Ovulation & Pregnancy (4.288).

### 3.2. Behavior Change

Twenty-five women downloaded and used the included apps for the assessment of behavior change.  Table 3 shows the demographic and clinical characteristics of the study participants.  Table 4 shows the results of behavioral change assessment for each of the included apps utilizing section G of the MARS app assessment tool. Period Tracker Flo, Ovulation Calendar & Pregnancy had the highest impact on changing behavior while Nyra-Period, Fertility & Ovulation Tracker App, and Maya-Period, Fertility, Ovulation & Pregnancy had the least.

## 4. Discussion

To the best of our knowledge, this study is the first to comprehensively review commercially available period tracker mobile apps and to independently evaluate their quality using a validated rating scale: the MARS expert rating scale. The current results show a widespread use of period tracking apps by Android and Apple users, with some apps reaching as high as 10,000,000+ downloads. Star ratings given to the surveyed applications by users ranged from 1.8 to 5. Meanwhile, the average quality scores obtained by utilizing the MARS rating scale ranged from 1.98 to 4.48.

Around one in seven people are estimated to use a smartphone globally, making mobile applications a vital part of everyday living [21]. This is true for health and well-being, where mobile applications have proved useful in enhancing health outcomes and promoting healthy behaviors, making patients an integral part of healthcare in the process [21, 22]. The use of gynecology and fertility apps has been previously reported. Symul et al. assessed the data of around 200,000 users of two period tracking apps to detect tracking behavior [23]. Another study found a few period tracking applications to be accurate in predicting cycle length and fertility window [24]. Most users were middle-aged and used the apps for Fertility Awareness Method (FAM) tracking and family planning. About 40% of the assessed cycles had a daily recording frequency, making these apps useful for the evaluation of ovulation and menstrual health. Furthermore, the mHealth app quality criteria included in the MARS recommend that a number of “app-specific” items are added to obtain information on the perceived impact of the app on the user's knowledge, attitudes, and intentions related to the target health behavior and improving user outcomes [20]. The app quality total score and four app-quality subscales had high internal consistencies, indicating that MARS provides a reliable indicator of overall app quality, as well as the quality subscales of the app engagement, functionality, aesthetics, and information quality. The exclusion of the subjective quality subscale from the overall mean app quality score, due to its subjective nature, strengthens the objectivity of the MARS tool as a measure of app quality. Moglia et al. rated period tracking applications based on their accuracy in cycle prediction and correctness of provided health information [25]. Twenty apps were assessed, most of which provided education on conception and contraception. A myriad of features was offered by the majority of apps, including tracking of cycle and symptoms, alerts, and information about cycle length. However, a few apps contained cited information or involved professionals.

The evidence behind health applications may be a subject of concern, as many of the apps claiming to be “evidence-based” were not in fact tested in trials. Instead, they were based on scientifically proven methods that may not necessarily be effective when embedded in an app design [21]. For example, Moglia et al. found a few period tracking apps that employed FAM for tracking ovulation and plan pregnancy [25]. The current study found that only a few of the available period tracking apps were based on scientific evidence. For example, fertility education and medical management (FEMM) was developed based on a comprehensive women's health program by the Reproductive Health Research Institute (RHRI), which collaborated with FEMM to develop a protocol focused on reproductive endocrinology [26]. Furthermore, CycleBeads, an app developed by the Institute for Reproductive Health at Georgetown University, was shown to be over 95% effective in efficacy trials [27]. The app is scientifically proven as a method for planning or preventing pregnancy by simply tracking the period in each cycle. It, thus, serves as an effective, easy to use natural birth control option. Similarly, the Dot, an app developed by Cycle Technologies in collaboration with experts in the fields of reproductive health and data science, was clinically tested and found to be 95% effective with typical use and 99% effective with perfect use for preventing and planning pregnancy and tracking cycles [28, 29]. Ovia fertility, an app aiming to reduce maternity costs and improve clinical outcomes, was also clinically assessed [30]. However, the remainder of the retrieved period tracking apps were not tested for efficacy or based on evidence, which presents a hurdle to the adoption of these apps by healthcare providers. The risk of bias is also present when apps are assessed by founders or funding bodies, as is the case with the app Natural Cycle, which was evaluated in three different studies [31–33]. Furthermore, the privacy and security of personal health information stored or transmitted by such apps must be ensured before healthcare providers can give the thumbs up for their use [34]. Other barriers to effective employment of mobile health apps in health sectors include hidden costs and requirement for Internet access. Despite the fact that most features of the included apps were offered for free, with limited to no in-app purchases, certain apps like Menstrual Calendar only offered free trials, while others required Internet access for certain or all functions. This may add hidden costs to these apps that could potentially mislead users, as such apps claim to be free. For example, Nyra-Period, Fertility & Ovulation Tracker App, and the highest scoring app, Nabta cycle, are both available for free and contain no in-app purchases but require Internet access to function.

On the other hand, factors contributing to the success of mHealth apps include ease of use and navigation, individualization, and a user-friendly design [5, 35]. In recent studies, mobile health apps have shown success in changing or improving health-related behaviors and overall health outcomes. Certain apps may use behavior change theories, such as the theory of planned behavior, to achieve targeted behavioral change [6, 35, 36]. In this study, we assessed the impact of certain period tracking apps by surveying 25 women who were asked to use these apps. Period Tracker Flo, Ovulation Calendar & Pregnancy showed the highest impact on health behaviors such as help-seeking and awareness of how and why users should insert their own targets. It also helped increase knowledge and understanding of women's own conditions during monthly periods. Similarly, Nabta cycle showed a high score in help-seeking and behavioral change. Femometer-Fertility Tracker and Nyra-Period, Fertility & Ovulation Tracker App offered a vast array of health-related articles, thus increasing the knowledge regarding different topics, including periods. On the other hand; Maya-Period, Fertility, Ovulation & Pregnancy feedback recorded the lowest score in most behavior change subquestions. However, the use of the objective MARS item provides a high level of reliability obtained beyond simple star rating, by offering a useful multidimensional tool for app assessment.

## 5. Conclusion

In general, mHealth apps offer a potentially cost-effective solution to symptoms monitoring, mood tracking, medications reminder, and promotion of patient engagement in their care and can enhance interactive care and communication with users with the same condition for healthcare providers.

These findings suggest that apps can be almost readily adopted into routine tracking and management and need further development to support comprehensive symptoms management for users to conduct accurate results. One of the ongoing priorities for engagement of mHealth apps into health care will be the strict assessment of app quality as demonstrated in this study. It is, thus, recommended that health professionals, including dietitians, health researchers, app developers, and maternal health experts, work in collaboration to design high-quality and evidence-based ovulation tracker apps. Improving the ability of apps to engage is also a targeted area for future improvement.

## Figures and Tables

**Table 1:** Description of the included apps.

App name	Numbers of downloads	Version	Star rating	Last updated
Nabta Cycle	100+	1.2.0	5	29 March 2019

Nyra-Period, Fertility & Ovulation Tracker App	100,000+	3.1	3.6	10 August 2019

Maya-Period, Fertility, Ovulation & Pregnancy	5,000,000+	3.6.7.7	4.8	3 September 2019

Period Tracker Flo, Ovulation Calendar & Pregnancy	10,000,000+	4.30.0	4.8	1 September 2019

Ovia Fertility: Ovulation & Period Tracker	1,000,000+	Varies with device	4.7	13 August 2019

Femometer-Fertility Tracker	100,000+	3.9.0 (3207)	4.7	3 September 2019

Fertility, Ovulation App & Pregnancy Tracker	500,000+	3.2	4.2	28 February 2019

Ladytimer Ovulation & Period Calendar	5,000,000+	4.5.9	4.8	5 June 2019

Period Tracker & Ovulation Calendar by PinkBird	500,000+	1.16.0	4.8	22 August 2019

Premom Ovulation Calculator	100,000+	1.6.3.2	4.3	27 August 2019

My Calendar-Period Tracker	100,000,000+	Varies with device	4.9	12 August 2019

Ovulation Calculator Fertility	100,000+	1.4.2	4.5	3 April 2017

Period Tracker and Women Diary	500,000+	6.0.1	4.7	13September 2018

Magic Girl Teen Period Tracker	100,000+	1.0.8	4.3	25 August 2018

Glow: Fertility Calculator and Ovulation Tracker	1,000,000+	7.8.1	4.7	29 August 2019

PRENSES KADIN—Period Calendar Ovulation Tracker	10,000+	1.0	3.9	13 October 2017

Period Tracker Cherry-Menstrual Cycle Tracker	50,000+	2.7.7	4.3	19 Nov2018

Spot On Period, Birth Control, & Cycle Tracker	500,000+	2.9.1	4.3	15 April 2019

My Cycles Period and Ovulation	1,000,000+	Varies with device	4.3	1 April 2019

FEMM Health Period and Ovulation Tracker	100,000+	1.1.882	4.0	31 May 2019

Period Tracker Mia Fem: Ovulation Calculator	1,000,000+	1.40	4.8	28 August 2019

Pepapp-Period, PMS, Ovulation Tracker	1,000,000+	3.4.1	4.4	31 July 2019

Simple Cycle	1,000+	1.3	4.6	14 August 2018

Pslove Period Tracker: PMS & Ovulation Calendar	50,000+	1.11.0	4.2	22 August 2019

Ovy-NFP, Period, Ovulation, bbt, Menstruation	50,000+	2.0.4	3.7	30 July 2019

Period Diary	100,000+	Varies with device	4.4	4 September 2019

Period Tracker-Period Calendar Ovulation Tracker	100,000,000+	Varies with device	4.9	12 August 2019

Pslove Period Tracker: PMS & Ovulation Calendar	50,000+	1.11.0	4.2	22 August 2019

Eve Period Tracker-Love, Sex & Relationships App	1,000,000+	2.14.6	4.6	5 September 2019

Once -A Special Period Tracker	1,000,000+	4.9.2	4.5	7 July 2019

MyPeriod Tracker	1,000+	1.2.2	No enough rating	30 March 2016

My Days-Ovulation Calendar & Period Tracker ™	5,000,000+	3.0.5	4.0	4 September 2019

Period Tracker	10,000,000+	Varies with device	4.7	31 August 2019

Ovulation-Fertility Tracker Eveline Cycle Calendar	5,000+	2.0.0	3.7	30 July 2019

Fertility Friend Ovulation App	1,000,000+	11.11	4.7	28 August 2019

Period Tracker Calendar & Ovulation Calculator	50,000+	1.2	4.3	6 February 2019

Clue Period Tracker-Ovulation and Cycle Calendar	10,000,000+	5.20.1	4.8	3 September 2019

My Menstrual Diary	1,000,000+	3.3	4.3	4 May 2016

Natural Cycles-Birth Control App	500,000+	3.2.12	4.5	16 August 2019

WomanLog Period Tracker & Calendar	5,000,000+	5.7.8	4.5	4 September 2019

Woman Log & Ovulation Tracker	10,000+	2.0.1	4.1	20 December 2016

CycleBeads Period & Ovulation	500,000+	2.30	4.6	4 December 2018

Menstruation Fertility Pro Lte	500,000+	3.0.9	4.1	3 June 2019

Kindara Fertility & Ovulation	100,000+	3.9.0	3.2	31 May 2019

Dot Period Tracker & Fertility Tracker	100,000+	1.195	4.3	27 August 2019

Flogirl Period	5,000+	1.2.0	4.5	8 August 2019

DaysyView	10,000+	2.3.0	1.8	3 September 2019

My Fertility Charts	100,000+	3.1.0	3.0	14 July 2019

OvaGraph—Official TCOYF App	10,000+	3.1.8	4.5	23 August 2019

**Table 2:** Mobile Application Rating Scale scores.

App name	Quality reviewer 1	Quality reviewer 2	Average quality	Subjective reviewer 1	Subjective reviewer 2
Nabta Cycle	4.45	4.5	4.475	3.75	5

Femometer-Fertility Tracker	4.7125	4.15	4.43125	4.25	4.25

Period Tracker Flo, Ovulation Calendar & Pregnancy	4.575	4.26	4.4175	4.25	4.5

Nyra-Period, Fertility & Ovulation Tracker App	4.37	4.37	4.37	4	4

Maya-Period, Fertility, Ovulation & Pregnancy	4.275	4.3	4.2875	2.75	4.25

Period Tracker & Ovulation Calendar by PinkBird	4.575	3.97	4.2725	4.25	3.2

Ovulation Calculator Fertility	4.6875	3.8	4.24375	4.25	3.75

Ovia Fertility: Ovulation & Period Tracker	4.075	4.22	4.1475	4.25	4

Premom Ovulation Calculator	4.125	3.9	4.0125	3.5	3.75

Eve Period Tracker-Love, Sex & Relationships App	4.7625	3.23	3.99625	4.25	2.5

Ladytimer Ovulation & Period Calendar	3.7375	4.12	3.92875	2.75	4

Fertility, Ovulation App & Pregnancy Tracker	3.625	4.12	3.8725	2.25	4

Simple Cycle	4.3125	3.4	3.85625	4	4

Glow: Fertility Calculator and Ovulation Tracker	3.875	3.7	3.7875	3.5	3.75

My Calendar-Period Tracker	3.6875	3.84	3.76375	2.25	3

Pslove Period Tracker: PMS & Ovulation Calendar	4.075	3.4	3.7375	3	3

Period Tracker and Women Diary	3.65	3.8	3.725	3	3.75

Period Tracker MIA Fem: Ovulation Calculator	3.9375	3.48	3.70875	3.25	2.5

Magic Girl Teen Period Tracker	3.55	3.7	3.625	1.75	2.75

PRENSES KADIN-Period Calendar Ovulation Tracker	3.575	3.66	3.6175	2.25	3

Period Tracker Cherry-Menstrual Cycle Tracker	3.5	3.64	3.57	2	2.5

Period Tracker-Period Calendar Ovulation Tracker	3.8625	3.27	3.56625	2.25	2.25
Once -A Special Period Tracker	3.9	3.22	3.56	2.25	2.5

FEMM Health Period and Ovulation Tracker	3.5875	3.5	3.54375	3	3

Pepapp-Period, PMS, Ovulation Tracker	3.625	3.4	3.5125	3.25	3

Ovy-NFP, Period, Ovulation, bbt, Menstruation	3.65	3.32	3.485	2.75	3.25

Pslove Period Tracker: PMS & Ovulation Calendar	3.25	3.4	3.325	2.75	2.75

Spot on Period, Birth Control, & Cycle Tracker	3.0125	3.6	3.30625	2	3

Clue Period Tracker—Ovulation and Cycle Calendar	3.7925	2.82	3.30625	3.5	2

Flogirl Period	4.25	2.33	3.29	3.5	1.75

Fertility Friend Ovulation App	3.6375	2.9	3.26875	2.25	2

My Cycles Period and Ovulation	2.95	3.5	3.225	2	4

Kindara Fertility & Ovulation	3.9125	2.5	3.20625	4	1.5

Period Tracker	3.325	3.05	3.1875	2.5	2.5

My Days-Ovulation Calendar & Period Tracker™	3.25	3.1	3.175	2.25	2.25

Period Diary	3	3.3	3.15	2	2.5

WomanLog Period Tracker & Calendar	3.575	2.66	3.1175	2.25	1.25

Ovulation Calendar App	3.1	3.3	3.2	1.75	1.25

MyPeriod Tracker	3	3.12	3.06	2	2.5

Woman Log & Ovulation Tracker	3.325	2.66	2.9925	2.5	1.25

Dot Period Tracker & Fertility Tracker	3.3625	2.48	2.92125	2.25	1

Period Tracker Calendar & Ovulation Calculator	2.9125	2.88	2.89625	1.75	2

CycleBeads Period & Ovulation	3.0875	2.58	2.83375	1.75	1.25

Ovulation-Fertility Tracker Eveline Cycle Calendar	2.65	3	2.825	2	2.25

My Menstrual Diary	2.725	2.79	2.7575	1.25	2.25

My Fertility Charts	3.125	2.27	2.6975	2.25	2

Menstruation Fertility Pro Lte	2.5	2.57	2.535	1	1

DaysyView	2.2625	2.3	2.28125	1	1.25

OvaGraph—Official TCOYF App	1.95	2	1.975	1	1

**Table 3:** Demographic and clinical characteristics of the participants.

Characteristics	*n* (%)
Age, years (range)	18–39

Marital status	

Single	19 (76)

Married	6 (24)

Highest education	

College graduate	11 (44)

Postgraduate studies	14 (56)

Occupation	

Unemployed/housewife	14 (56)

Employed	11 (44)

Platform	

Android	15(60)

iPhone	10(40)

Period status	

Regular	23 (92)

Irregular	2 (8)

Goal of using period tracker app	

Tracking periods	25 (100)

Avoiding pregnancy	2 (8)

Understanding own body	2 (8)

Length of menstrual cycle	

24–27	4 (16)

28	15 (60)

29–40	6 (24)

Premenstrual symptoms	

Breast pain and tenderness	19 (76)

Mood changes	17 (68)

Fatigue	19 (76)

Bloating	13 (52)

Weight gain	8 (32)

Acne	12 (48)

Sleep disturbances	7 (28)

Appetite changes	14 (56)

**Table 4:** Behavioral change questions—section G MARS.

App	Awareness	Knowledge	Attitudes	Intention to change	Help-seeking	Behavior change
Nabta Cycle	3.6	3.6	3.8	3.8	4	4

Femometer-Fertility Tracker	3.4	3.4	3.8	3.6	3.6	3.2

Period Tracker Flo, Ovulation Calendar & Pregnancy	4.2	4.2	4	4	4	4

Nyra-Period, Fertility & Ovulation Tracker App	3.4	3.8	3.2	3.6	3.6	3.2

Maya-Period, Fertility, Ovulation & Pregnancy	3.4	3.4	3.4	3.4	3.4	3.8

## Data Availability

The data used to support the findings of this study are available from the corresponding author upon request.
